# Sodium‐glucose cotransporter 1/2 inhibition and risk of neurodegenerative disorders: A Mendelian randomization study

**DOI:** 10.1002/brb3.3624

**Published:** 2024-07-15

**Authors:** Jinxin Liu, Xinxiu Shi, Yankun Shao

**Affiliations:** ^1^ Department of Neurology China–Japan Union Hospital of Jilin University Changchun Jilin Province China

**Keywords:** drug target, hemoglobin A1c, Mendelian randomization, neurodegenerative disorders, sodium‐glucose cotransporter

## Abstract

**Introduction:**

This study aims to evaluate the effects of sodium‐glucose cotransporter 1 inhibitors (SGLT1i) and sodium‐glucose cotransporter 2 inhibitors (SGLT2i) on neurodegenerative disorders and to investigate the role of hemoglobin A1c (HbA1c) levels.

**Methods:**

Utilizing drug target Mendelian randomization, we employed single nucleotide polymorphisms (SNPs) proximal to the SLC5A1 and SLC5A2 genes to analyze the influence of SGLT1i and SGLT2i on Alzheimer's disease (AD), Parkinson's disease (PD), multiple sclerosis (MS), frontotemporal dementia (FTD), Lewy body dementia (LBD), and amyotrophic lateral sclerosis (ALS), with type 2 diabetes (T2D) as a positive control. An additional analysis examined the impact of HbA1c levels on the same disorders.

**Results:**

SGLT1i exhibited a significant association with decreased risk for ALS and MS. Conversely, SGLT2i were linked to an increased risk of AD, PD, and MS. Elevated HbA1c levels, independent of SGLT1 and SGLT2 effects, were associated with an increased risk of PD. Sensitivity analyses supported the robustness of these findings.

**Conclusion:**

Our study suggests that SGLT1i may confer protection against ALS and MS, whereas SGLT2i could elevate the risk of AD, PD, and MS. Additionally, elevated HbA1c levels emerged as a risk factor for PD. These findings underscore the importance of personalized approaches in the utilization of SGLT inhibitors, considering their varying impacts on the risks of neurodegenerative diseases.

## Graphical abstract

The impact of SGLT1 inhibitors and SGLT2 inhibitors on neurodegenerative disorders and the role of blood glucose levels.

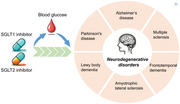

## 1 INTRODUCTION

Neurodegenerative disorders manifest through a gradual loss of neurons in the central nervous system (CNS), which in turn impairs specific brain functions. Such conditions are widely categorized based on their clinical presentations, with cognitive or behavioral disorders and extrapyramidal and pyramidal movement disorders being the most common (Dugger & Dickson, 2017). While some neurodegenerative disorders like Alzheimer's disease (AD) and Parkinson's disease (PD) are common, others including amyotrophic lateral sclerosis (ALS), multiple sclerosis (MS), frontotemporal dementia (FTD), and Lewy body dementia (LBD) are rare. It is estimated that neurodegenerative diseases will affect 131.5 million patients worldwide by 2050, posing a substantial socioeconomic burden (Tofaris & Buckley, 2018). By altering or inducing the expression of specific proteins, gene therapy may not only protect or restore neurons but potentially correct underlying disease mechanisms (Sudhakar & Richardson, 2019). Current therapies for neurodegenerative disorders, however, aim at symptomatic relief and do not address the underlying pathology. The lack of drugs specifically targeted to neurodegenerative disorders has generated great interest in repurposing existing medications to slow neurodegeneration and reduce mortality.

Sodium–glucose cotransporters 1 (SGLT1, encoded by SLC5A1 gene) and sodium–glucose cotransporters 2 (SGLT2, encoded by SLC5A2 gene) play key roles in epithelial glucose reabsorption, with SGLT2 retrieving most glucose in the tubular system of the kidney and SGLT1 in the intestine reabsorbing the remainder (Rieg & Vallon, 2018). Inhibitors of SGLT1 and SGLT2 represent novel therapeutic agents for type 2 diabetes mellitus (T2D), with SGLT2 inhibitors like dapagliflozin, canagliflozin, and empagliflozin now widely used in clinical practice due to their durable glucose lowering, fewer adverse effects, and additional cardiorenal benefits (Cui et al., 2023; Scheen, 2020). Dual SGLT1/2 inhibitors like sotagliflozin have also displayed favorable pharmacokinetics and effective glycemic control in healthy subjects and T2D subjects (Rosenstock et al., 2015). Emerging evidence suggests that SGLT1 inhibitors (SGLT1i) and SGLT2 inhibitors (SGLT2i) might impact neurodegenerative processes. Imaging using 4‐FDG (fluoro‐4‐deoxy‐D‐glucopyranoside) revealed the presence of SGLTs in multiple regions of the CNS in several isoforms, particularly in the cerebellum, hippocampus, frontal cortex, parietal cortex, caudate nucleus, putamen, amygdala, and paraventricular nucleus of the hypothalamus (Rizzo et al., 2022). The SGLT2 inhibitor empagliflozin exhibited neuroprotection in a PD rodent model by restricting oxidative stress and restoring antioxidant mechanisms (Ahmed et al., 2022). Recent studies have increasingly highlighted the neuroprotective effects of SGLT2 inhibitors. Population‐based studies suggest that SGLT2 inhibitors are associated with a decreased risk of dementia and depression (Liebers et al., 2023; Wu et al., 2023). Preclinical experiments have shown that these medications can improve brain energetics (Pawlos et al., 2021), and a recent randomized controlled trial demonstrated improved mood outcomes with the selective SGLT2 inhibitor empagliflozin in combination with citalopram (Zandifar et al., 2024). These findings suggest that SGLT2 inhibitors may have a broader therapeutic potential beyond their glycemic control effects. SGLT1 is also brain‐expressed and has been reported to have a strong connection with AD pathogenesis (Szablewski, 2021). By mediating SGLT1, GLP1 exhibited neuroprotective effects in the hippocampus, given that SGLT1 downregulation could result in reduced neuron glucose metabolism and heightened neurodegeneration (Mei et al., 2023). However, research on SGLT1 and SGLT2 roles in neurodegeneration remains limited, and their precise contributions across neurodegenerative disorders are likely inconsistent and require further elucidation.

Mendel's second law of inheritance stipulated the random distribution of genetic material during gamete formation. This shuffling evenly segregated confounders across genotypes. Mendelian randomization (MR) exploited this trait to model the influence of modifiable exposures on outcomes, by utilizing genetic variants as proxies for exposures not biased by confounding (Bandres‐Ciga et al., 2020). This instrumental variable approach circumvented issues plaguing conventional observational analyses, providing robust causal inference without relying on experimental intervention. Drug target MR analysis leveraged only variants in or near the encoding gene of a therapeutic target. Through creating an instrument that mimicked a therapeutic intervention and conducting regression analysis, this type of MR study evaluated whether modulating an exposure causally influenced disease (Burgess et al., 2017; Ference, 2022). Valid instrumental variables (IVs) for MR analysis met three key assumptions: (1) robust association with the exposure (relevance); (2) independence from confounders (validity); and (3) impacts on outcome mediated only through the exposure (exclusion restriction) (Davies et al., 2018). We applied drug target MR to determine whether genetically predicted inhibition of SGLT1 and SGLT2 affected the risk of neurodegenerative disorders. Using the recently published genome‐wide association study (GWAS) summary‐level data, we created IVs for SGLT1/2 inhibition. We investigated if these instruments associated with AD, PD, MS, ALS, FTD, LBD, and T2D, implying causal effects of SGLT1/2 manipulation on neurodegenerative outcomes. An additional analysis also evaluated the effect of HbA1c levels on the same disorders.

## 2 MATERIALS AND METHODS

**FIGURE 1:**
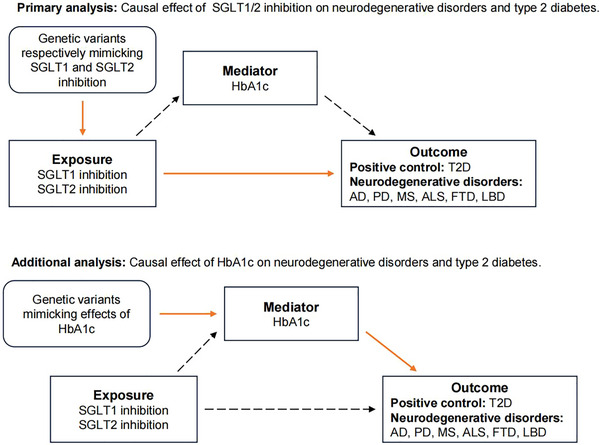
Study design. Diagram of the two steps of MR models: primary analysis, to establish the causal effect of SGLT1/2 inhibition on neurodegenerative disorders and T2D, and additional analysis, to establish the causal effect of the HbA1c on neurodegenerative disorders and T2D. SGLT, sodium‐glucose cotransporter; HbA1c, hemoglobin A1c; AD, Alzheimer's disease; PD, Parkinson's disease; MS, multiple sclerosis; ALS, amyotrophic lateral sclerosis; FTD, frontotemporal dementia; LBD, Lewy body dementia; T2D, type 2 diabetes.

This MR study comprised primary and additional analyses, with the study design outlined in Figure 1, following the STROBE‐MR guidelines.

### 2.1 Selection of SGLT1 and SGLT2 instrumental variables

The summary data of hemoglobin A1c (HbA1c) included 344,182 European individuals from the MRC Integrative Epidemiology Unit (IEU) open genome‐wide association study (GWAS) project (Lyon et al., 2021). To derive instrumental variables (IVs) targeting SGLT1 and SGLT2 to lower HbA1c, we selected single nucleotide polymorphisms (SNPs) located within ± 500 kb of the SLC5A1 gene and associated with HbA1c at genome‐wide significance (*p* < 5 × 10^−7^) for SGLT1 inhibition. For SGLT2 inhibition, SNPs within ± 1000 kb of the SLC5A2 gene and associated with HbA1c at genome‐wide significance (*p* < 5 × 10^−8^) were chosen. By setting these thresholds, we ensured selection of a sufficient number of SNPs accurately representing HbA1c levels. Subsequently, SNPs with linkage disequilibrium within 100 kb genomic distance (*r* ^2^ > 0.3) were clumped using the 1000 Genome European reference panel (1000 Genomes Project Consortium et al., 2015). We quantified the statistical power of genetic variants by estimating the strength of each SNP using *F* statistics with the formula: *F* = *R* ^2^(*N*−2)/(1−*R* ^2^). Another formula was used to calculate *R* ^2^ for the SNP instrument: (2 × eaf × (1 – eaf) × beta^2^)/[(2 × eaf × (1 – eaf) × beta^2^) + (2 × eaf×(1 – eaf) × *N* × se^2^)] (Papadimitriou et al., 2020). SNPs failing to meet specific conditions were excluded: (1) minor allele frequency (MAF) < 0.01 (Chen et al., 2023); (2) *F*‐statistic < 10 (Pierce et al., 2011); (3) not presented in the outcome GWAS; (4) *p*‐value < 5 × 10^−5^ in the outcome GWAS (to fulfill the exclusion restriction: the IVs should not be directly associated with the outcome) (Baumeister et al., 2020). Ultimately, 8 significant SNPs for SGLT1 and 18 significant SNPs for SGLT2 were obtained (Table S1).

### 2.2 Source of outcomes

Seven diseases served as outcomes for the drug target MR analysis, with T2D as a positive control dataset and the other six as primary outcomes, all derived from the European population. The T2D dataset comprised 57,698 cases and 308,252 controls obtained from GWAS summary statistics in the FinnGen study (release 9) (Kurki et al., 2023). Summary datasets for PD, MS, AD, and FTD were also sourced from the FinnGen study (release 9). Summary statistics for ALS and LBD were downloaded from the NHGRI‐EBI GWAS Catalog on 29/09/2023 for study GCST90027164 and GCST90001390 (Chia et al., 2021; Sollis et al., 2023; van Rheenen et al., 2021). Detailed information on exposure and outcome GWAS summary statistics is provided in Table S2. Methods for HbA1c assessment and diagnostic criteria for diseases can be accessed on their respective source websites. As data for each exposure and outcome sample were derived from different European cohorts, there was minimal sample overlap.

### 2.3 Statistical analysis

SGLT2 inhibitors (e.g., Canagliflozin, Dapagliflozin) have been widely employed in treating T2D, with numerous studies indicating SGLT1 as a therapeutic target in diabetes (Gyimesi et al., 2020; Song et al., 2016). Utilizing the summary data from the GWAS of T2D as a positive control for outcomes, we validated the efficacy of the IVs of SGLT1 and SGLT2 inhibitors. After aligning the exposure‐related IVs with the outcome datasets, IVW (inverse variance weighted) served as the primary method for MR analysis. Weighted median and MR Egger were additionally applied to test the robustness of the main IVW estimates against horizontal pleiotropy (Zhao et al., 2023). Heterogeneity testing was conducted using MR Egger and IVW, with Cochran's Q statistic calculated to assess IV heterogeneity, where *p* > .05 indicated no significant heterogeneity. To evaluate potential bias and horizontal pleiotropy from ineffective IVs, the MR‐Egger intercept test was performed (Burgess & Thompson, 2017). The MR‐PRESSO (Mendelian randomization pleiotropy residual sum and outlier) method was utilized to detect and correct horizontal pleiotropy by removing outliers and testing for significant differences in causal estimates before and after outlier removal (Verbanck et al., 2018). MR analysis was rerun post‐outlier removal as detected by the MR‐PRESSO test. Leave‐one‐out analysis was conducted to assess the impact of outlying and pleiotropic SNPs on causal estimates, sequentially omitting each SNP to identify any driving associations. MR analysis was executed using R (version 4.3.1) with the TwoSampleMR (version 0.5.7) packages (Hemani et al., 2018).

### 2.4 Additional analysis

Our primary analysis encompassed only variants proximal to the SLC5A1 or SLC5A2 gene, acting as proxies for the effect of SGLT1/2 inhibitor drugs, providing an adequate estimate of their impact on neurodegenerative disorders. To delve further into the pathway through which SGLT1/2 inhibitors act on neurodegenerative disorders, we explored associations between genetically predicted HbA1c (independent of SGLT1 and SGLT2) and neurodegenerative disorders. HbA1c was instrumented using variants genome‐wide significantly associated with HbA1c (*p* < 5 × 10^−8^), excluding those within ± 500 kb of SLC5A1 and ± 1000 kb of SLC5A2, and clumped for linkage disequilibrium within 10,000 kb (*r* ^2^ > 0.001). This stringent criterion was chosen as variants were selected genome‐wide rather than from a single gene locus. These variants were anticipated to affect HbA1c independently of SGLT1/2, potentially indicating a causal relationship between blood sugar levels and the risk of neurodegenerative disorders. MR analyses including IVW and weighted median were conducted as part of the additional analysis.

To enable a more comprehensive assessment of the robustness of our research methodology, we additionally analyzed the effects of SGLT1/2 inhibitors on cardiovascular diseases (CVD) and chronic kidney disease (CKD).

## 3 RESULTS

### 3.1 Positive control analysis

Consistent with expectations, the IVW and weighted median methods demonstrated that SGLT1i and SGLT2i significantly reduced T2D risk (SGLT1i: OR_IVW = 0.47, 95%CI_IVW = 0.33− 0.67, P_IVW = 2.13 × 10^−5^; SGLT2i: OR_IVW = 0.81, 95%CI_IVW = 0.66− 0.99, P_IVW = 0.04).

### 3.2 Primary analysis: Effect of inhibition of SGLT1 and SGLT2 on risk of neurodegenerative disorders

**FIGURE 2:**
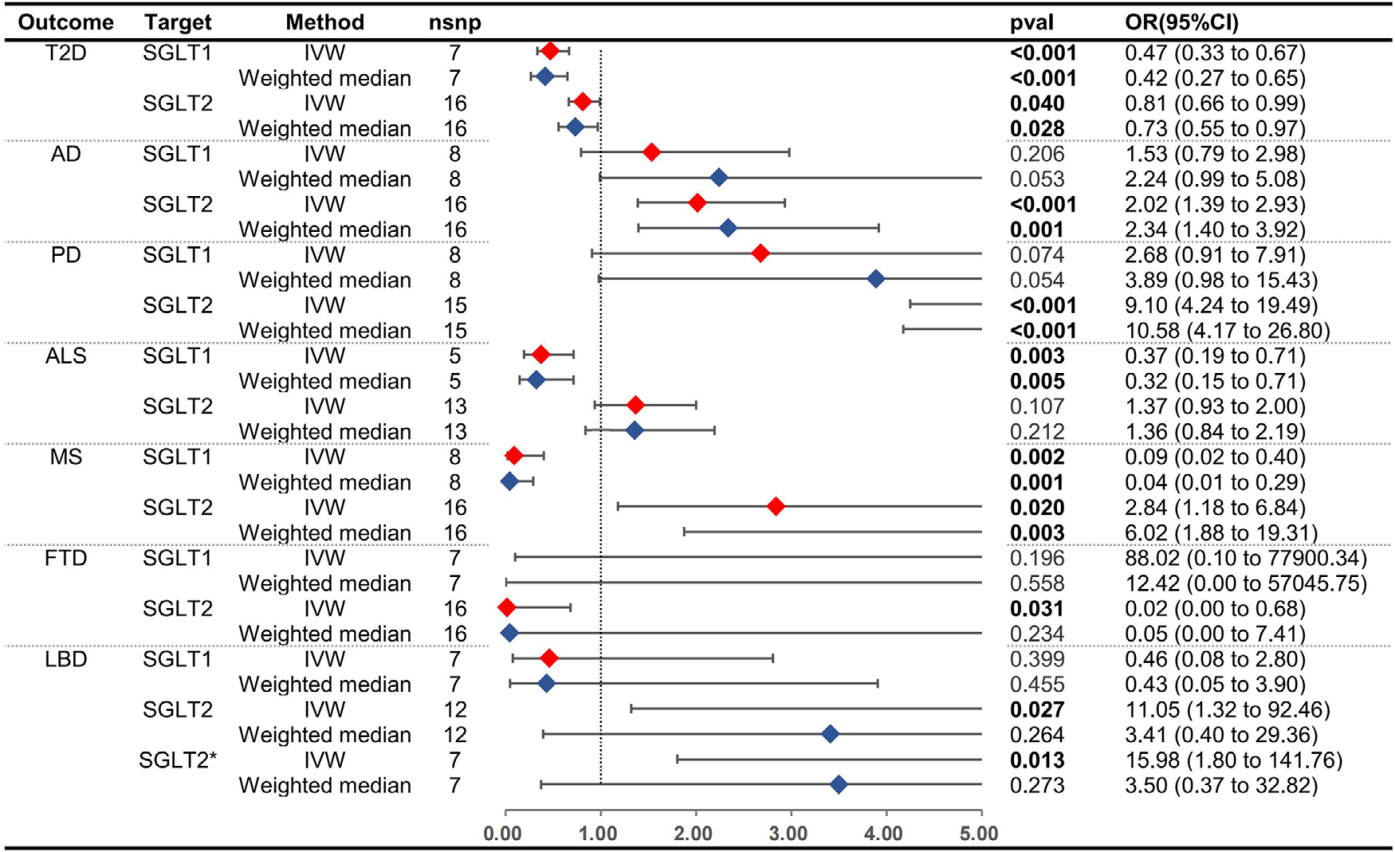
MR estimates and forest plot of the effect of SGLT1/2 inhibition on neurodegenerative disorders and T2D. Asterisk (*) represents the linkage disequilibrium parameter in the selection of IVs changes from *r* ^2^ < 0.3 to *r* ^2^ < 0.1. nsnp, number of single nucleotide polymorphisms; pval, *p* value; OR, odds ratio; CI, confidence Intervals; HbA1c, hemoglobin A1c; AD, Alzheimer's disease; PD, Parkinson's disease; MS, multiple sclerosis; ALS, amyotrophic lateral sclerosis; FTD, frontotemporal dementia; LBD, Lewy body dementia; T2D, type 2 diabetes; SGLT, sodium‐glucose cotransporter; IVW, inverse variance weighted.

The effect estimates of the MR analyses for the causal association of SGLT1i and SGLT2i with six neurodegenerative disorders and T2D are depicted in Figure 2 and Table S3. The MR analyses indicated that SGLT1i exhibited a significant protective effect on ALS and MS (ALS: IVW OR = 0.37, 95%CI = 0.19− 0.71, *p* < .01. MS: IVW OR = 0.09, 95%CI = 0.02− 0.40, *p* < .01), with no significant associations observed for other neurodegenerative disorders (AD: P_IVW = 0.21. PD: P_IVW = 0.07. FTD: P_IVW = 0.20. LBD: P_IVW = 0.40). For SGLT2i, significant deleterious effects were observed on AD, PD, and MS (AD: IVW OR = 2.02, 95%CI = 1.39− 2.93, *p* < .01. PD: IVW OR = 9.10, 95%CI = 4.24−19.49, *p* < .01. MS: IVW OR = 2.84, 95%CI = 1.18− 6.84, *p* = .02), while no significant associations were found with ALS, LBD, or FTD (ALS: P_IVW = 0.11. LBD: P_IVW = 0.01, weighted median *p* = .27; FTD: P_IVW = 0.03, weighted median *p* = .23). Several sensitivity analyses, including heterogeneity testing, MR‐Egger intercept testing, MR‐PRESSO (Table S4), and leave‐one‐out analysis (Figure S1, Figure S2), were performed. Initial results showed significant heterogeneity and pleiotropy in the causality between SGLT2i and PD, with rs8050500 identified as a potential outlier. After outlier removal, no significant heterogeneity or pleiotropy was observed, although the increased risk of PD with SGLT2 inhibition remained significant. Similar analyses were conducted for LBD, initially indicating significant heterogeneity and pleiotropy, but without identifying individual outliers. By employing stricter SNP selection criteria and removing an outlier (rs28692853), no significant heterogeneity or pleiotropy was observed, and no causal link between SGLT2i use and LBD risk was found. Sensitivity analyses revealed no evidence of heterogeneity or horizontal pleiotropy for other outcomes (*p* > .05), with negligible effects observed in leave‐one‐out analyses for T2D and neurodegenerative disorders.

### 3.3 Additional analysis

Using 319 SNPs to proxy HbA1c, the additional analysis demonstrated that higher HbA1c levels significantly increased the risk of T2D and PD, with no associations observed for other neurodegenerative disorders. Detailed results of the MR analysis and sensitivity analyses are provided in Table S5. The effects of SGLT1/2 inhibitors on CVD and CKD are presented in Table S6 and Figure S3, wherein SGLT2 inhibitors exhibited a protective effect against CVD.

## 4 DISCUSSION

In the context of emerging treatments for neurodegenerative disorders and type 2 diabetes, the role of sodium‐glucose cotransporter 2 inhibitors (SGLT2i) and sodium‐glucose cotransporter 1 inhibitors (SGLT1i) has gained significant attention. Our drug target MR analysis has brought to light intriguing associations between these treatments and the risk of neurodegenerative diseases, sparking discussions on their therapeutic potentials and risks.

### 4.1 Differential impact on neurodegenerative disorders

The investigation into the effects of SGLT1 and SGLT2 inhibitors on neurodegenerative disorders has yielded strikingly divergent outcomes. The observed protective effects of SGLT1i against ALS and MS underscore the need to delve into the biological underpinnings of these findings. The substantial reduction in risk for these diseases suggests that SGLT1 inhibition may influence pathways critical to their pathophysiology. The exact mechanisms remain elusive; however, the neuroprotective potential of SGLT1i may be linked to the modulation of glucose metabolism in the CNS, which is crucial for neuronal health and function. Inhibiting SGLT1 enhances glucose homeostasis through decreased absorption of dietary glucose in the intestine and elevated secretion of gastrointestinal incretins such as glucagon‐like peptide‐1 (Song et al., 2016). This mechanism could potentially alleviate abnormal glucose metabolism in neurodegenerative diseases like ALS and MS, though our additional analysis did not find an effect of high blood sugar on neurodegenerative disorders, except for PD.

Moreover, riluzole, a drug used to treat ALS, can suppress glutamate release, thereby preventing glutamate‐induced activation of sodium channels on postsynaptic neurons, subsequently reducing excitotoxicity (Lin et al., 2015). This suggests that the modulation of neurotransmitter levels may be a therapeutic pathway of SGLT1i. Additionally, mizagliflozin, a selective SGLT1 inhibitor, improved vascular cognitive impairment by preventing hippocampal neuronal death in a mouse model, suggesting its potential to cross the blood–brain barrier (Ishida et al., 2021). Furthermore, an electrophysiological study in mice demonstrated expression of both SGLT2 and SGLT1 in rostral ventrolateral medulla (RVLM) bulbospinal neurons. Inhibition of these SGLT transporters suppressed RVLM neuron activity, implying that pharmacological inhibition of SGLT2 and SGLT1 may produce antihypertensive effects by inhibiting sympathetic nervous system activity through suppression of RVLM neuronal activity (Rahman & Nishiyama, 2024). However, previous studies have also demonstrated that SGLT1 has a protective effect on the nervous system, complicating the role of SGLT1 further. During a metabolic deficit, the uptake of glucose is increased by greater SGLT function, which is essential during epileptic seizures (Melo et al., 2016). An experiment on rats demonstrated that higher glucose levels following pilocarpine‐induced status epilepticus aggravated SGLT1 translocation in the hippocampus. Directly infusing glucose into the hippocampus, potentially via glucose sensors T1R2/T1R3, elevated SGLT1 expression in hippocampal subfields linked to neuroprotection (de Melo et al., 2021).

Conversely, SGLT2i appears to have a detrimental association with AD, PD, and MS, as indicated by odds ratios suggesting a significant increase in risk. This highlights a potential risk for the exacerbation of these conditions with SGLT2i use. Despite the benefits of SGLT2i in glycemic control and cardiovascular outcomes (Packer, 2022), their influence on neurodegenerative processes might be complex and potentially deleterious. Nevertheless, the harmful mechanisms of SGLT2 inhibitors in the mentioned diseases are not clearly outlined and appear to be overshadowed by research focusing on their therapeutic potential. Detailed research and clinical data would be required to thoroughly understand any adverse mechanisms. Notably, two SGLT2 inhibitors, canagliflozin and dapagliflozin, have been linked to the adverse event of diabetic ketoacidosis (DKA) (Pelletier et al., 2021). During DKA, warning signs of cerebral edema can include neurological changes like restlessness, irritability, increased drowsiness, and paresis or paralysis of cranial nerves (Szmygel et al., 2016). Moreover, a significantly increased risk of hypoglycemic events was reported in patients using canagliflozin along with metformin and sulfonylureas (Pelletier et al., 2021). Hypoglycemia can lead to a wide array of nonspecific neurological manifestations including confusion, seizures, focal neurological deficits, stupor, and sometimes coma. Seizures and other neurological deficits may also present during hypoglycemia (Barbara et al., 2017). In contrast to the current research findings, some studies indicate that SGLT2i may provide neuroprotective effects by improving mitochondrial function and antioxidative effects, potentially benefiting the control of PD progression (Lin et al., 2021; Tang et al., 2023).

Our findings, which indicate an increased risk of AD, PD, and MS with SGLT2 inhibition, stand in contrast to the growing evidence supporting the neuroprotective effects of these inhibitors. This paradox may be explained by the dual inhibitory activity of SGLT2 inhibitors, many of which also inhibit SGLT1 to some extent (Zhao et al., 2023). The relative selectivity of these inhibitors could play a critical role in their overall neuroprotective versus neurodegenerative effects. Therefore, we propose that future research should focus on comparing the neuroprotective effects of various SGLT2/1 inhibitors to their relative selectivity.

Additionally, there is a need for real‐world data to evaluate whether individuals with PD on SGLT2 inhibitors experience any worsening of the disease course. Given that low HbA1c levels have been shown to be protective, it is essential to understand whether the observed increased risk in our study translates into clinical practice. Such research would provide invaluable insights and guide clinical decision‐making.

We have observed several trends that did not meet a significance threshold but could still be biologically relevant. For example, while the associations between SGLT1 inhibitors and AD and PD were not statistically significant, the direction of the effect suggests a potential detrimental role of SGLT1 inhibitors. Similarly, the trends observed for SGLT2 inhibitors in relation to FTD indicate possible protective effects, though these associations did not reach statistical significance. We propose that these trends should be investigated further in larger and more diverse cohorts to determine their validity. The exploration of these initial signals is crucial for identifying potential therapeutic targets and understanding the complex mechanisms underlying neurodegenerative diseases. We recommend future studies to apply more stringent statistical thresholds and replication in other datasets to confirm these preliminary findings.

The distinct outcomes observed for SGLT1 and SGLT2 inhibition on different neurodegenerative disorders underscore the complexity of these conditions and the intricate interplay between metabolic regulation and neurodegeneration.

### 4.2 Additional analysis linking HbA1c levels with PD

Our findings from additional analysis confirmed that HbA1c levels are causally associated with an increased risk of PD. This has been clinically confirmed by several cohort studies demonstrating that diabetes mellitus is associated with an increased risk of PD (Jeong et al., 2020). Elevated blood sugar, often a consequence of diabetes, leads to an increase in the production of harmful compounds known as advanced glycation end‐products (AGEs). These AGEs have the ability to bind to receptors known as RAGE (Receptor for Advanced Glycation End‐products). The interaction between AGEs and RAGE can trigger inflammatory pathways known to contribute to the development of various diseases, including neurodegenerative disorders. The AGE‐RAGE axis is suggested to play a significant role in the pathogenesis of these diseases by promoting oxidative stress and inflammation, which can lead to cellular damage and disease (Bhattacharya et al., 2023). Combined with the results of our additional analysis above, the process by which SGLT2 inhibitors increase the risk of PD may have additional mechanisms other than lowering blood sugar levels. Given that we did not observe evidence of an overall effect of HbA1c (not caused by SGLT1 and SGLT2) on neurodegenerative disorders except for PD, the causal association of SGLT1i on ALS and MS, and SGLT2i on AD and MS, is unlikely to be related to glycemic control, and other mechanisms specific to SGLT1/2 may be driving the association.

### 4.3 Role of SGLT1/2 inhibitors in CVD and CKD

Our analysis also revealed that SGLT2 inhibitors have a significant protective effect on CVD, aligning with clinical evidence of their cardioprotective benefits through improved glycemic control, blood pressure reduction, and beneficial myocardial effects (Frąk et al., 2023). However, SGLT1 inhibitors showed no significant association with CVD. While numerous laboratory studies have demonstrated the potential cardioprotective effects of SGLT1 inhibitors, clinical evidence from large‐scale trials is still lacking to conclusively validate their therapeutic benefits for cardiovascular health (Li & Xu, 2022). For CKD, neither SGLT1 nor SGLT2 inhibitors demonstrated a significant association, despite clinical trials showing nephroprotective benefits of SGLT2 inhibitors. The effects of SGLT1/2 inhibition on CKD progression might take a long time to manifest. MR studies typically capture lifelong genetic exposure, which may not reflect the shorter‐term impact of pharmacological inhibition. Literature indicates that SGLT2 inhibitors also carry the risk of adverse renal events, such as acute kidney injury and urinary tract infections (Stottlemyer et al., 2023). MR study reflects the combined effect of drugs on disease, so this duality complicates the overall risk assessment of SGLT2 inhibitors on renal health.

### 4.4 Clinical implications

The results of this study serve as a signpost to guide additional clinical research in this area. They suggest a need for a cautious approach when considering SGLT2i for patients at risk of or with existing neurodegenerative diseases. Conversely, the potential neuroprotective effects of SGLT1i could open new avenues for the treatment of ALS and MS, diseases that currently have limited therapeutic options.

### 4.5 Limitations and strengths

To our knowledge, this is the first study to use MR analysis to investigate the relationship between SGLT1/2 inhibition and neurodegenerative disorders in the general population. We also explored whether SGLT1/2 inhibitors affect neurodegenerative disorders through blood glucose levels. Furthermore, the inclusion of type 2 diabetes as a positive control outcome in the study design helped validate the reliability of the IVs. Our study is not without limitations. Firstly, the MR approach, while powerful, relies on several assumptions. The selected genetic instruments must affect the risk of disease solely through the pathway of interest, which may not always hold true. Despite rigorous selection criteria, there remains a possibility of pleiotropy, where genetic variants influence multiple traits. The significant heterogeneity and pleiotropy initially detected in the study highlight the complexities of the genetic architectures underlying these diseases. Secondly, our analysis is based on summary‐level data from European populations, which may limit the generalizability of our findings to other ethnic groups. The genetic architecture and environmental factors influencing neurodegenerative diseases can vary significantly across populations. Thirdly, the complex nature of neurodegenerative disorders and the multifactorial risk factors involved make it challenging to account for all potential confounders. While we employed various sensitivity analyses, including MR‐Egger and MR‐PRESSO, to detect and adjust for pleiotropy, some residual confounding might still exist. Additionally, our study does not provide direct evidence of causation but rather suggests potential associations that need to be validated in clinical settings. The genetic variants used as proxies for SGLT1/2 inhibition reflect lifelong exposure, which might differ from the effects of pharmacological inhibition over a shorter duration. Therefore, MR analysis is more useful for determining the potential direction of a causal effect rather than quantifying its size. Finally, this research is an exploratory study that aims to find as many potentially positive things as possible and guide future research rather than to confirm specific associations. Therefore, multiple testing corrections were not applied to avoid increasing the risk of type II errors. We emphasize the need for replication of these findings in independent datasets and suggest that the observed trends should be interpreted with caution. Future studies should validate these associations using larger cohorts and more stringent statistical approaches.

## 5 CONCLUSION

In conclusion, our study provides valuable insights into the dichotomous roles of SGLT inhibitors. SGLT1i exhibited a significant protective effect against ALS and MS, suggesting potential neuroprotective properties that warrant further investigation. Conversely, SGLT2i were associated with an increased risk of AD, PD, and MS, highlighting the need for cautious use in patients at risk for these neurodegenerative diseases. Additionally, elevated HbA1c levels were independently associated with an increased risk of PD, indicating that blood sugar control is crucial in managing neurodegenerative disease risks. These findings underscore the importance of personalized approaches in the utilization of SGLT inhibitors, considering their varying impacts on different neurodegenerative diseases. Further research is imperative to unravel the precise mechanisms at play and to validate these findings in clinical settings. Such studies should focus on diverse populations and employ more stringent methodologies to confirm the potential benefits and risks associated with SGLT1 and SGLT2 inhibition.

## AUTHOR CONTRIBUTIONS

**Jinxin Liu**: Conceptualization; writing—original draft; writing—review and editing; resources; methodology. **Xinxiu Shi**: Software; data curation; formal analysis. **Yankun Shao**: Conceptualization; data curation; project administration.

## CONFLICT OF INTEREST STATEMENT

The authors declare that they have no competing interests.

### PEER REVIEW

The peer review history for this article is available at https://publons.com/publon/10.1002/brb3.3624.

## Supporting information

Supporting Information

## Data Availability

All data generated or analyzed during this study are included in this published article and its supplementary information files.
