# Supplementary material for: Sodium‐glucose cotransporter 1/2 inhibition and risk of neurodegenerative disorders: A Mendelian randomization study
Source: Brain Behav. 2024 Jul 15;14(7):e3624. doi: 10.1002/brb3.3624 (PMC11250420; doi:10.1002/brb3.3624)
Supplement: Supplementary file 1 — Supporting Information [file BRB3-14-e3624-s001.docx]

**Supplementary Materials**

**Abbreviation**

nsnp, number of single nucleotide polymorphisms; pval, p value; OR, odds ratio; CI, confidence Intervals; SGLT, sodium-glucose cotransporter; HbA1c, hemoglobin A1c; AD, Alzheimer's disease; PD, Parkinson's disease; MS, multiple sclerosis; ALS, amyotrophic lateral sclerosis; FTD, frontotemporal dementia; LBD, Lewy body dementia; T2D, type 2 diabetes; CVD, cardiovascular disease; CKD, chronic kidney disease; IVW, inverse variance weighted; MR-PRESSO, Mendelian randomization pleiotropy residual sum and outlier; EA, effect allele; OA, other allele.

**Note:**

In **Table S3** and **Table S4**, Asterisk (*) represents the linkage disequilibrium parameter in the selection of IVs changes from r^2^<0.3 to r^2^<0.1.

**Table S1.** Instrumental variables of SGLT1 inhibitor and SGLT2 inhibitor.


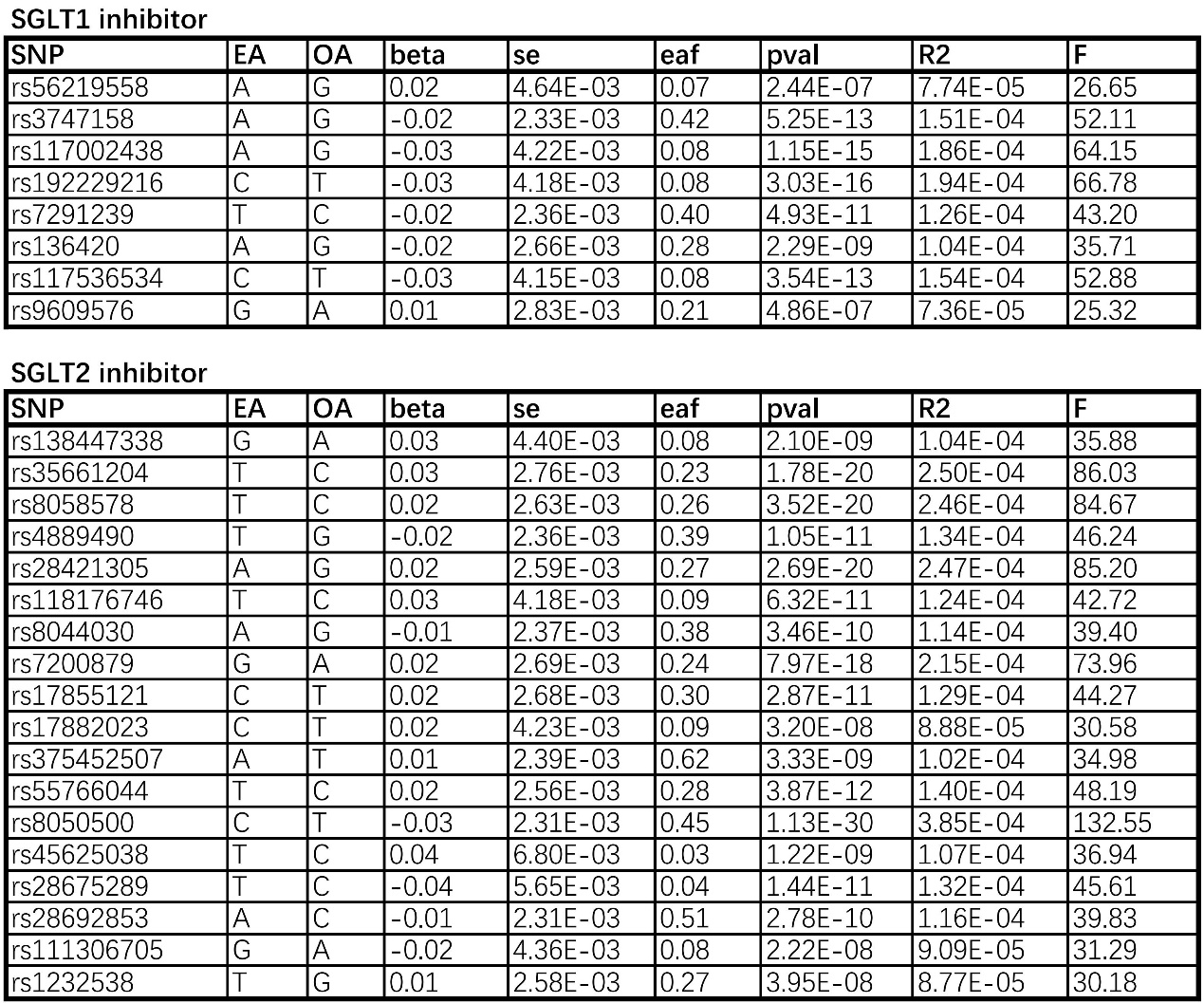


**Table S2.** Data source used in Mendelian randomization analysis. (In the FinnGen study, the phenotype of cardiovascular diseases encompasses a range of disorders, including hypertensive diseases, ischemic heart diseases, pulmonary embolism, cerebrovascular diseases, diseases of arteries, arterioles and capillaries, diseases of veins, lymphatic vessels and lymph nodes, other cardiovascular diseases, and other heart diseases.)


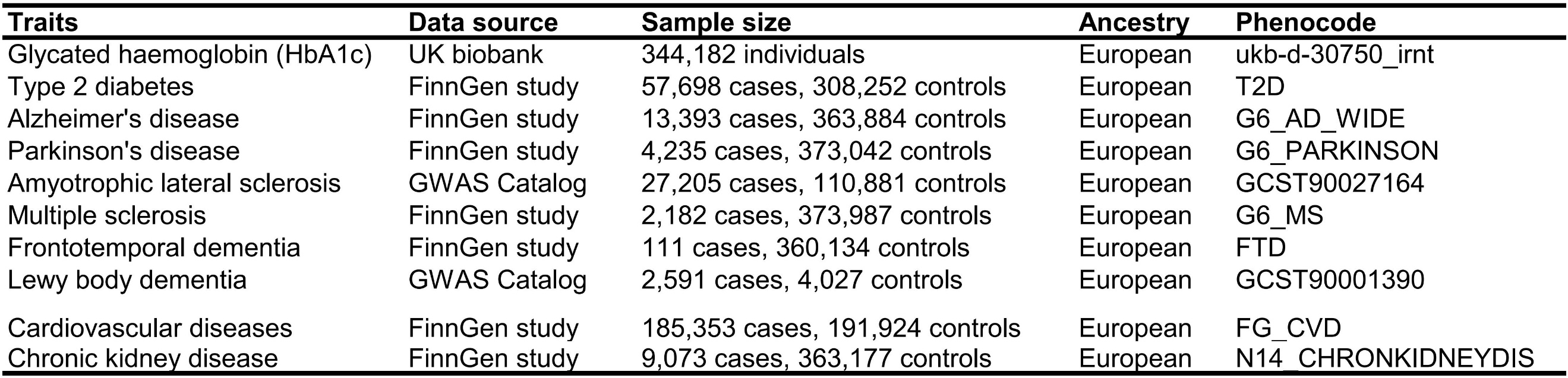


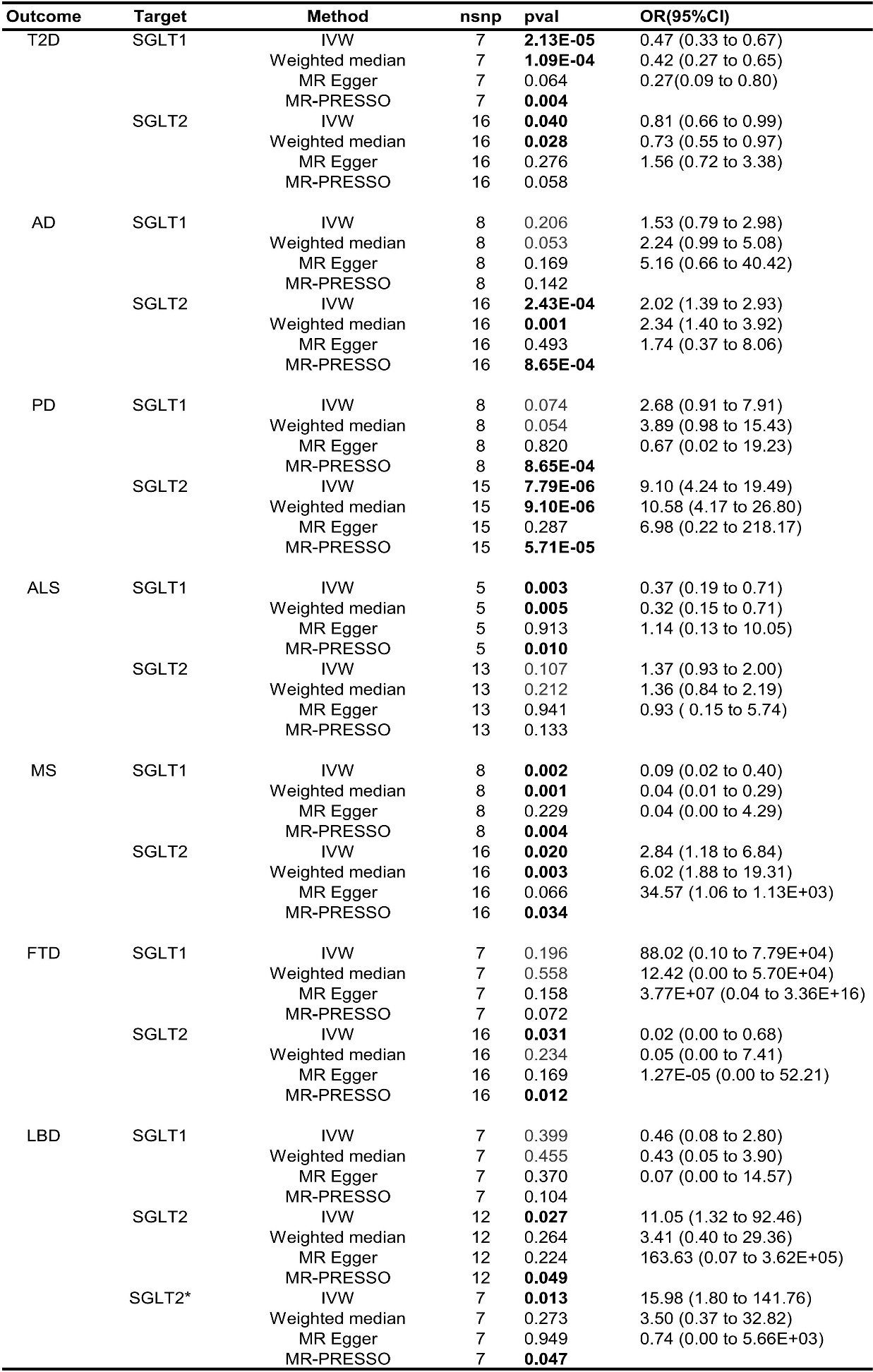
**Table S3.** MR estimates of the effect of SGLT1/2 inhibition on neurodegenerative disorders and T2D.


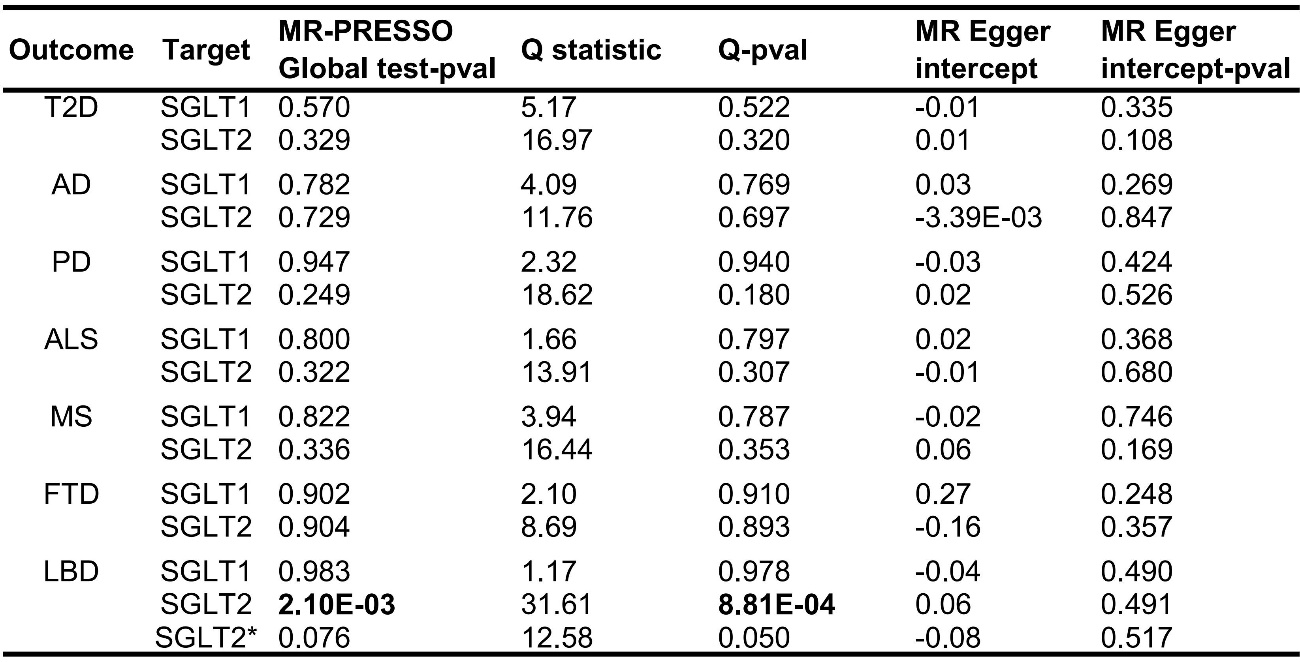
**Table S4.** The result of heterogeneity test and horizontal pleiotropic test of SGLT1/2 inhibition on neurodegenerative disorders and T2D.


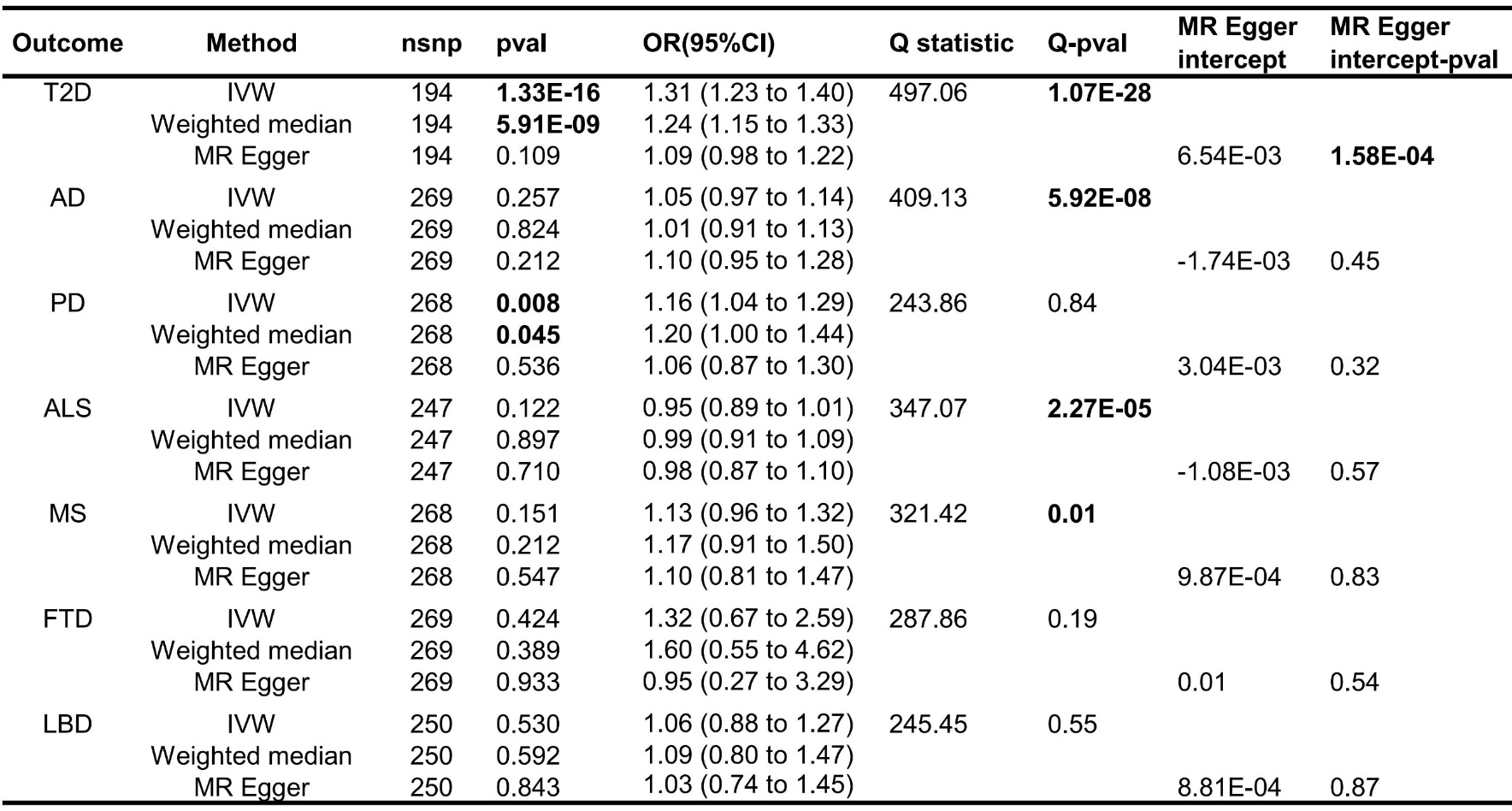
**Table S5.** MR estimates and sensitive analysis of the effect of HbA1c on neurodegenerative disorders and T2D.

**
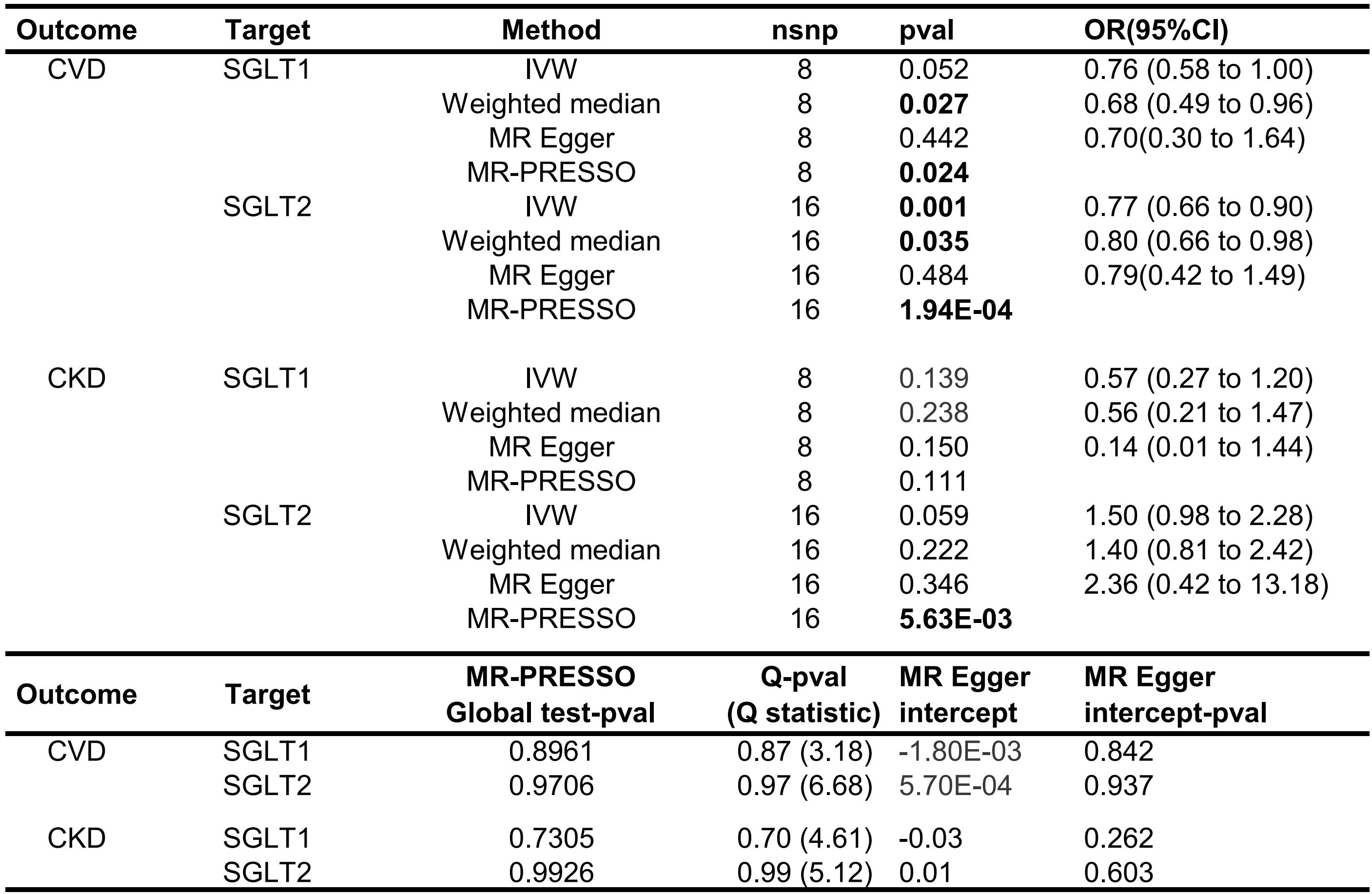
Table S6.** MR estimates and sensitive analysis of the effect of SGLT1/2 inhibitors on CVD and CKD.

*
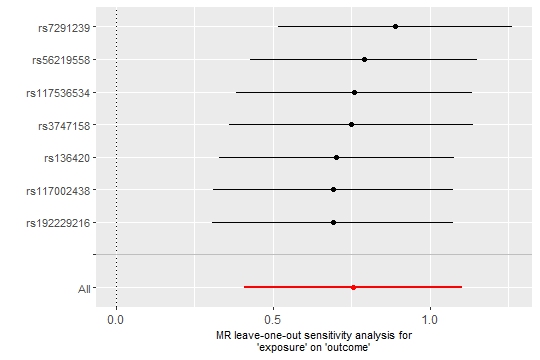
***Figure S1.** Leave-one-out analysis of SGLT1i on ***A.*** T2D, ***B.*** AD, ***C.*** PD, ***D.*** ALS, ***E.*** MS, ***F.*** FTD and ***G.*** LBD.

***A.***

*
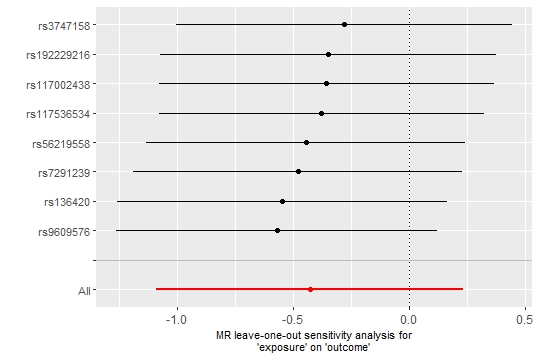
****B.***

*
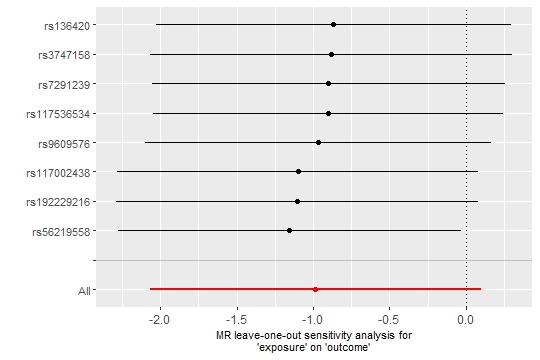
****C.***

*
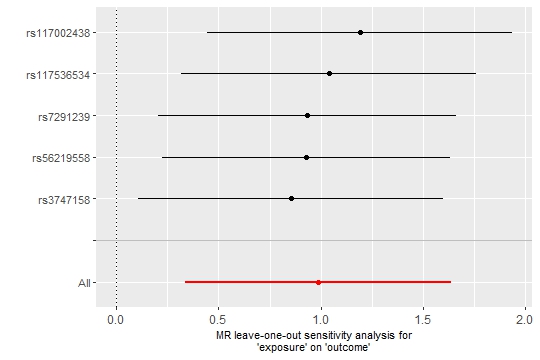
****D.***

*
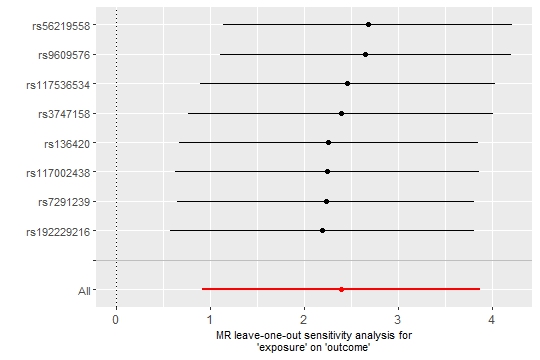
****E.***

*
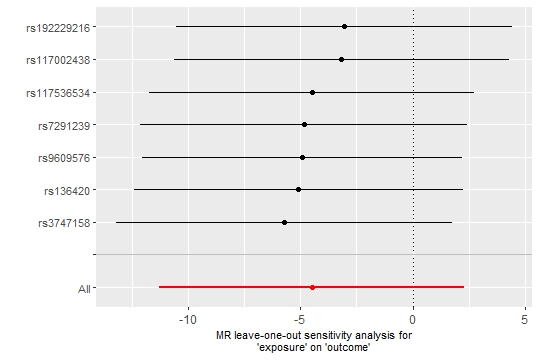
****F.***

*
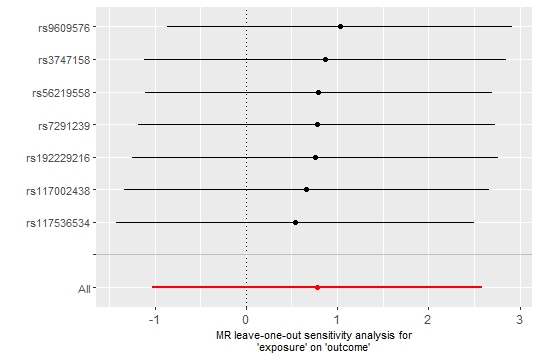
****G.***

**Figure S2.** Leave-one-out analysis of SGLT2i on ***A.*** T2D, ***B.*** AD, ***C.*** PD, ***D.*** ALS, ***E.*** MS, ***F.*** FTD, ***G.*** LBD and ***H.*** LBD (r²=0.1).


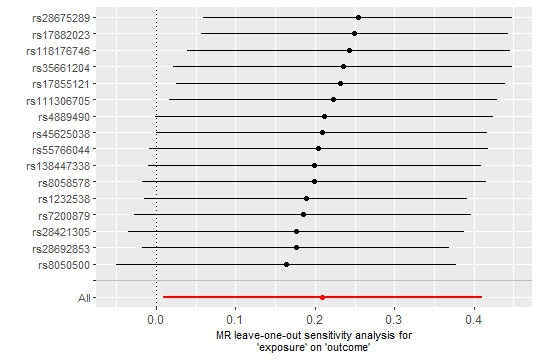
***A.***


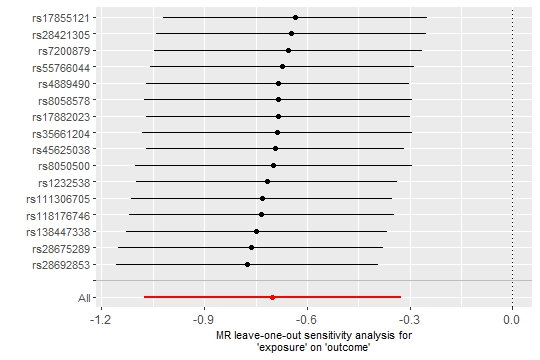
***B.***


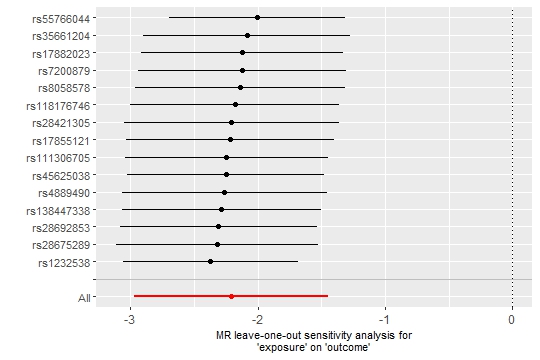
***C.***


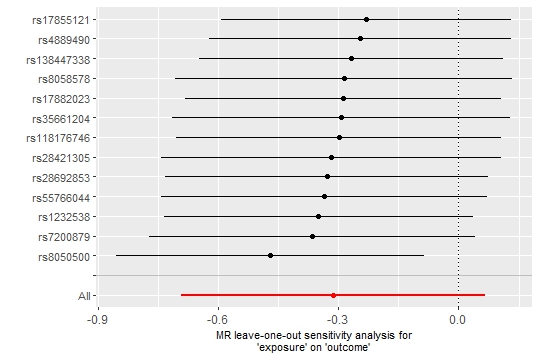
***D.***


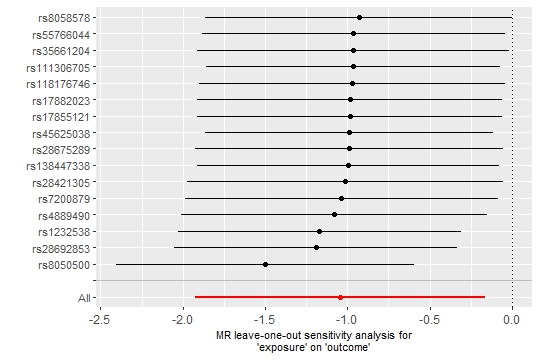
***E.***


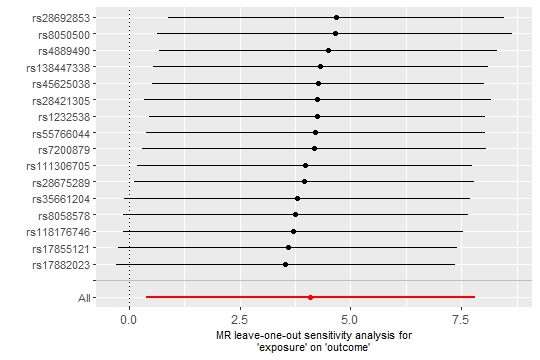
***F.***


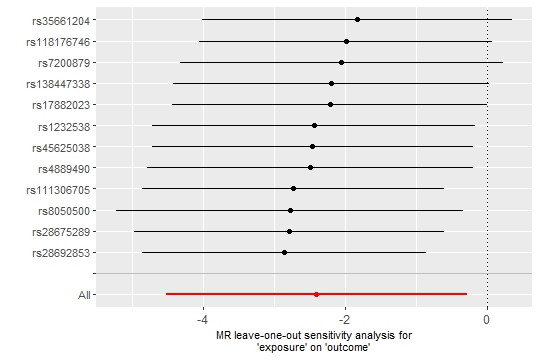
***G.***


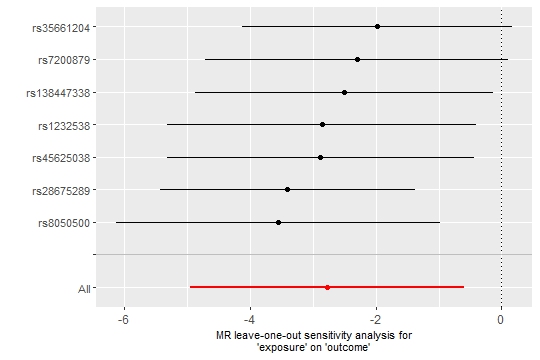
***H.***

**Figure S3.** Leave-one-out analysis of SGLT1i on ***A.*** CVD, ***B.*** CKD, and SGLT2i on ***C.*** CVD, ***D.*** CKD.

***
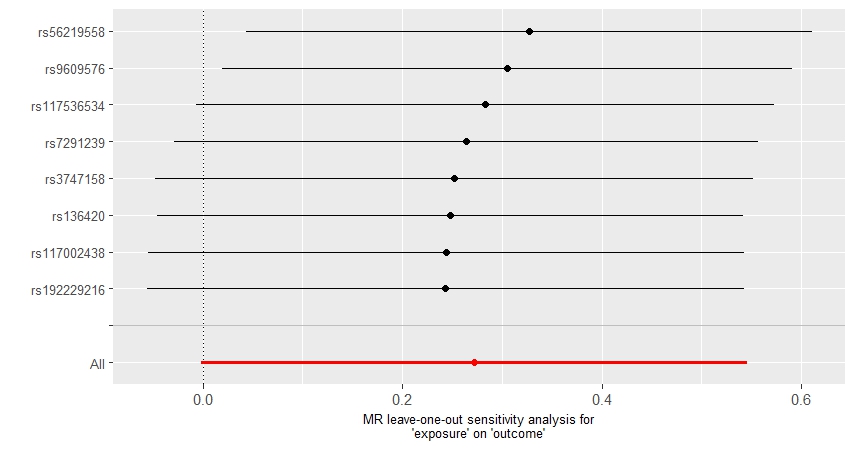
A.***

***
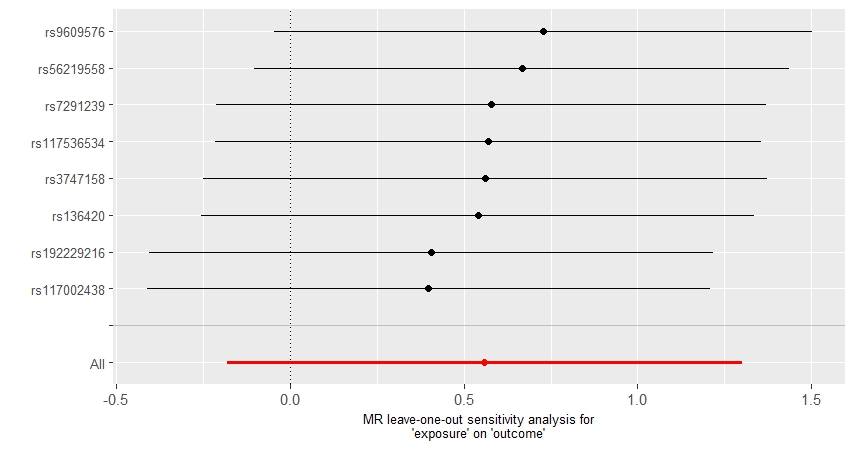
B.***

***
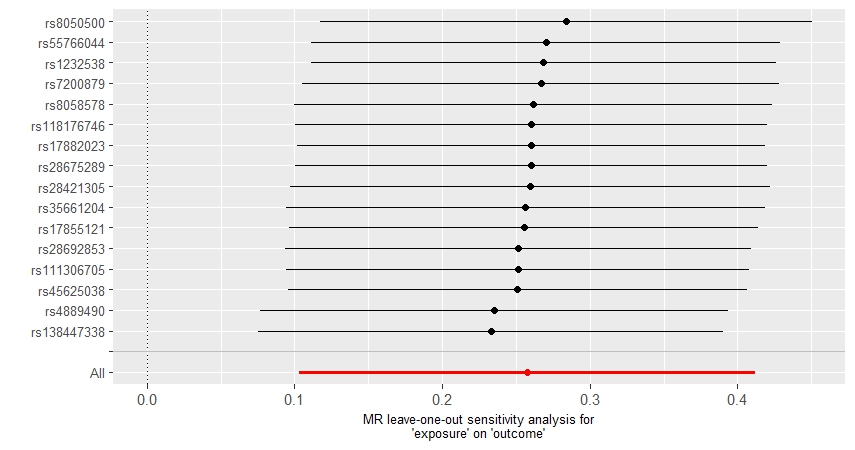
C.***

***
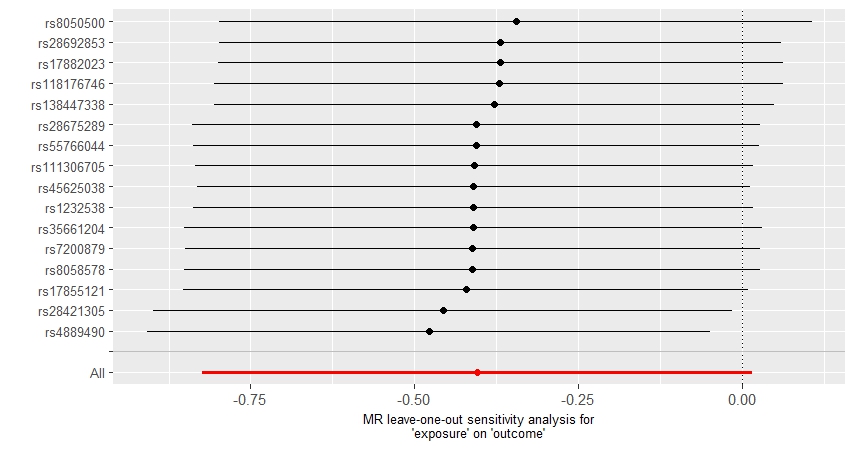
D.***
